# Refining the diagnostic suspicion of obstructive sleep apnea

**DOI:** 10.1055/s-0043-1776417

**Published:** 2023-11-08

**Authors:** Gilmar Fernandes do Prado

**Affiliations:** 1 Clínica Neuro-Sono, São Paulo, SP, Brazil.; 2 Universidade Federal de São Paulo, Escola Paulista de Medicina, Departamento de Neurologia e Neurocirurgia, São Paulo, SP, Brazil.

Patients with obstructive sleep apnea have various symptoms and clinical signs that allow the doctor, or even people close to them, to suspect the disease. Some suggestive clinical data can be taken in isolation or in association with other variables, and the careful and attentive doctor is expected to identify these assumptions of clinical value. 1 The classic profile of a patient with OSA is very variable, and a lot of information about the relevant aspects to consider now seems to be better known. Soon after its description and the possibility of a formal diagnosis (Polysomnography), clinical descriptions placed much more emphasis on obesity as an anatomical factor, daytime sleepiness as the main consequence, and loud snoring as a marker. Understanding the impact of obesity on the anatomy and dynamics of the airways has brought important information about the isolated effect of facial conformation (ethnically determined or not) which, added to obesity, strongly predisposes to sleep-disordered breathing. 2

There have been several epidemiological studies, until a more complex and refined phenotype of this very prevalent disease was reached, where anatomical aspects contribute, whether genetically determined or due to exposure to factors that hinder or prevent nasal breathing, with the presence of daytime sleepiness no longer being so relevant or necessary in the diagnostic grounds. Once we could identify the most relevant aspects, other suggestive medical variables or biomarkers are now showing up with the help of physicians and, of course, patients. 3

In this sense, the work of Sinem Nedime Sökücü and colleagues contributes to recognizing the broad spectrum of consequences attributable to OSA, and their data lends itself to alerting us and providing insights into possible dysfunctions underlying the clinical complex manifested by a patient. In a very conscientious and well-planned study, the authors excluded various confounding factors and analyzed data from a representative sample, culminating in demonstrating the role of the TG Index in the diagnostic suspicion of OSA, proving it to be an independent factor.

We know that the patient with OSA when in front of the doctor mostly provides many clinical indicators, obtained from the history and physical examination. Many complaints (low back pain, muscle tenderness, for example), for which other specialists are working to clarify, may represent some consequence of the poor quality of sleep promoted by obstructive sleep apnea (e.g., inflammation, arousal, oxygen desaturation, sleep fragmentation), and which fortunately disappear or improve when the patient is properly treated. 4

The paper published in this issue of Arquivos de Neuropsiquiatria (Triglyceride-glucose index as a predictor of obstructive sleep apnoea severity in the absence of traditional risk factors) 5 is an example of the care that physicians should offer to patients under their attention. Insulin resistance is associated with many comorbidities, promotes cognitive dysfunction, and plays a recognized role in the epidemiology of Alzheimer's disease. 6 7 Therefore, ruling out the possibility of a patient having a treatable disease such as OSAS, using the TG Index, considering the cutoff of 8.615 (another important piece of data from this study) is important for doctors in various specialties.

When referring to patients with OSAS, we must emphasize that after many years of experience, we are fully aware that we still have a lot to learn and clarify. However, some principles cannot slip through the neurologist's fingers. First, the diagnosis must be made through complete polysomnography, where classic sleep parameters are analyzed together with the usual physiological parameters, since the TG Index is associated with oxygen desaturation index, AHI, minimum O _2_ saturation, TTS with O2 below 90%, parameters that must be reliable, so that the doctor can work with the variability of laboratory and polysomnographic factors safely in his decisions. 8 Second, treatment with CPAP should be based on polysomnography to titrate the ideal positive pressure, i.e., the pressure that eliminates respiratory events, snoring, increased airway resistance, and micro-awakenings. Ideally, this should be done in the supine position and in REM sleep, by an experienced and welcoming professional, who should be able to choose the best mask to use with the patient, preferably a nasal mask. 9 Third, positive pressure treatment should be maintained throughout the entire night's sleep, or even during the day if the patient has a habit of dozing off.

The study by Sinem Nedime Sökücü and colleagues adds another piece of information to our understanding of this complex disease, alerting us to look out for metabolic events that may be determinants of known comorbidities and others that we have yet to recognize.
